# Localization and Mapping Using Only a Rotating FMCW Radar Sensor

**DOI:** 10.3390/s130404527

**Published:** 2013-04-08

**Authors:** Damien Vivet, Paul Checchin, Roland Chapuis

**Affiliations:** 1 Institut Pascal, Université Blaise Pascal, Clermont Université, BP 10448, F-63000 Clermont-Ferrand, France; E-Mails: damien.vivet@univ-bpclermont.fr (D.V.); roland.chapuis@univ-bpclermont.fr (R.C.); 2 Institut Pascal, CNRS, UMR 6602, F-63171 Aubière, France

**Keywords:** radar sensor, localization, mapping, distortion, Doppler, odometry

## Abstract

Rotating radar sensors are perception systems rarely used in mobile robotics. This paper is concerned with the use of a mobile ground-based panoramic radar sensor which is able to deliver both distance and velocity of multiple targets in its surrounding. The consequence of using such a sensor in high speed robotics is the appearance of both geometric and Doppler velocity distortions in the collected data. These effects are, in the majority of studies, ignored or considered as noise and then corrected based on proprioceptive sensors or localization systems. Our purpose is to study and use data distortion and Doppler effect as sources of information in order to estimate the vehicle's displacement. The linear and angular velocities of the mobile robot are estimated by analyzing the distortion of the measurements provided by the panoramic Frequency Modulated Continuous Wave (FMCW) radar, called IMPALA. Without the use of any proprioceptive sensor, these estimates are then used to build the trajectory of the vehicle and the radar map of outdoor environments. In this paper, radar-only localization and mapping results are presented for a ground vehicle moving at high speed.

## 1. Introduction

The increased autonomy of robots is directly linked to their capability to perceive their environment. Simultaneous Localization and Mapping (SLAM) techniques, which associate perception and movement, are particularly interesting because they provide advanced autonomy to vehicles such as robots. In outdoor environments, the localization and mapping tasks are more complex to achieve under various climatic constraints. In this context, classical sensors are limited because of the technologies used: ultrasound is perturbed by wind, optical sensors (laser, vision) by rain, fog or the presence of dust or by poor lighting conditions [1]. An analysis of the effects of such challenging conditions on perception and an identification of their strong links with common perceptual failures are presented in [2]. One of the particularities of this work is the use of a new microwave radar sensor, which returns both range and velocity information combined with received signal power information, reflected by the targets in the environment, observed with a 360° per second rotating antenna and with a range from 5 to 100 *m*. The long range and the robustness of radar waves to atmospheric conditions make this sensor well suited for extended outdoor robotic applications.

Range sensors are widely used for perception tasks but it is usually assumed that the scan of a range sensor is a collection of depth measurements taken from a single robot position [3]. This can be done when working with lasers that are much faster than radar sensors and can be considered instantaneous when compared with the dynamics of the vehicle. However, when the robot is moving at high speed, most of the time this assumption is unacceptable. Important distortion phenomena appear and cannot be ignored; moreover, with radar sensor, the movement of the sensor itself generates Doppler effect on the data. For example, in a radar mapping application [4], the sensor delivers one panoramic radar image per second. When the robot is going straight ahead, at a low speed of 5 *ms*^−1^, the panoramic image includes a 5-m distortion. In the case of a laser range finder with a 75 *Hz* scanning rate, distortion exists but is ignored. This assumption is valid for low speed applications, nevertheless still moving straight ahead at a speed of 5 *ms*^−1^, a 7*cm* distortion effect appears. At classical road vehicle speeds (in cities, on roads or highways) more important distortions can be observed. Of course, the rotation of the vehicle itself during the measurement acquisition is another source of disturbance that cannot be neglected for high speed displacement or with slow sensors. When the sensor is too slow, a “*stop & scan*” method is often applied [5].

Another contribution presented in this paper is to propose a full radar-based odometry, which does not use any proprioceptive sensor but only distortion formulation and Doppler velocity analysis. The estimation of a vehicle's displacement or ego-motion is a widely studied problem in mobile robotics. Most applications are based on proprioceptive data provided by odometer sensors, gyrometers, IMU or other positioning systems such as GPS [6]. However, in order to estimate motion, some research works tried to use only exteroceptive data. Thus, Howard [7], Kitt *et al.* [8] and Nistér *et al.* [9] proposed a visual odometry without proprioceptive data. Tipaldi and Ramos [10] proposed to filter out moving objects before doing ego-motion. In such an approach, exteroceptive ego-motion is considered as intended to augment rather than replace classical proprioceptive sensors. Sometimes, classical displacement measurements are much more difficult and have limitations: inertial sensors are prone to drift, and wheel odometry is unreliable in rough terrain (wheels tend to slip and sink) and as a consequence visual odometric approaches are widely studied [11–13]. For example, in an underwater or naval environment classical ego-motion techniques are not suitable. In [14], Elkins *et al.* presented localization system for cooperative boats. In [15], Jenkin *et al.* proposed an ego-motion technique based on visual SLAM fused with IMU. In order to find displacement with exteroceptive sensors such as range finders, the scan matching method is commonly used [16–18] but each scan is corrected with proprioceptive sensors especially when the sensor is slow. In all scan matching work, distortion is taken into account but considered as a disturbance and thus corrected.

The only work dealing with distortion as a source of information used a rolling shutter specific camera. In [19], Ait-Aider *et al.* computed instantaneous 3D pose and velocity of fast moving objects using a single camera image but, in their context, prior knowledge about the observed object is required. In mobile robotics, we have no *a priori* knowledge about the surrounding environment of the robot. To the best of our knowledge, there is absolutely no work in the field of mobile robotics literature considering distortion as a source of information in an odometric purpose.

The originality of this paper is to study and use data distortion and Doppler effect as sources of information in order to estimate the vehicle's displacement. The linear and angular velocities of the mobile robot are estimated by analyzing the distortion of the measurements provided by the mobile ground-based panoramic Frequency Modulated Continuous Wave (FMCW) radar, called IMPALA. Then, the trajectory of the vehicle and the radar map of outdoor environments are built. Localization and mapping results are presented for a ground vehicle application when driving at high speed.

Section 2 presents the microwave radar scanner developed by a Irstea research team (in the field of agricultural and environmental engineering research) [20]. Section 3 focuses on the analysis of the Doppler effect for velocimetry purpose. Section 4 gives the formulation of the principle used in order to extract information from the distortion. Finally, Section 5 shows experimental results of this work. Section 6 concludes.

## 2. The IMPALA Radar

The IMPALA radar was developed by the IRSTEA in Clermont-Ferrand, France, for applications in the environmental monitoring domain and robotics. It is a Linear Frequency Modulated Continuous Wave (LFMCW) radar [21]. The principle of a LFMCW radar consists in transmitting a continuous frequency modulated signal, and measuring the frequency difference (called beat frequency *F_b_*) between the transmitted and the received signals. One can show that *F_b_* can be written as:

(1) $$F_{b}=\underset{F_{r}}{\underset{︸}{\frac{4\Delta FF_{m}R}{c}}}+\underset{F_{d}}{\underset{︸}{\frac{2\dot{R}}{\lambda }}}$$

where Δ*F* is the frequency excursion, *F_m_* the modulation frequency, *c* the light velocity, λ the wavelength, *R* the radar-target distance (*R* < *R_max_*) and *Ṙ* the radial velocity of the target. The first part *F_r_* of Equation (1) only depends on the range *R*, and the second part *F_d_* is the Doppler frequency introduced by the radial velocity *Ṙ*.

The IMPALA radar is panoramic. It is a monostatic radar, *i.e.*, a common antenna is used for both transmitting and receiving. The rotating antenna achieves a complete 360° scan around the vehicle in one second, and a signal acquisition is realized at each degree. The maximum range of the radar is 100 m. The radar includes microwave components, electronic devices for emission and reception, data acquisition and signal processing unit.

Data acquisition and signal processing units are based on an embedded Pentium Dual Core 1.6 *GHzPC*/104 processor. Computed data is transmitted using an Ethernet link for visualization and further processing. Main characteristics of the radar are described in Table 1.

**Figure 1.**
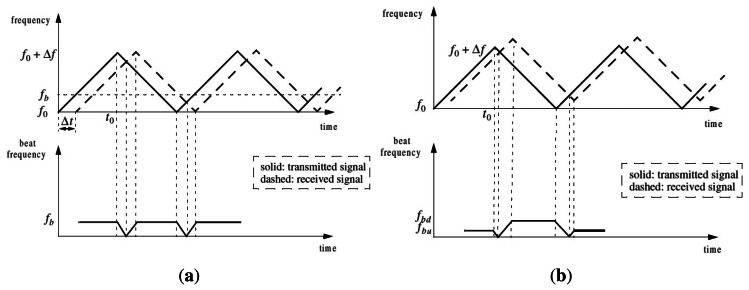
Triangular modulation with (**a**) static target; (**b**) mobile target.

In order to solve the distance-velocity ambiguity, a triangular modulation function is applied. In Figure 1(a), the full line represents the transmitted signal while the dashed line is the echo signal on a stationary target at a distance *R* of the sensor. The beat frequency *f_b_* is defined as the difference between the transmitted and the received wave:

$$f_{b}=f_{\text{reception}}-f_{\text{emission}}$$

Time shift Δ*t* is directly linked to the distance of the detected target.

**Figure 2.**
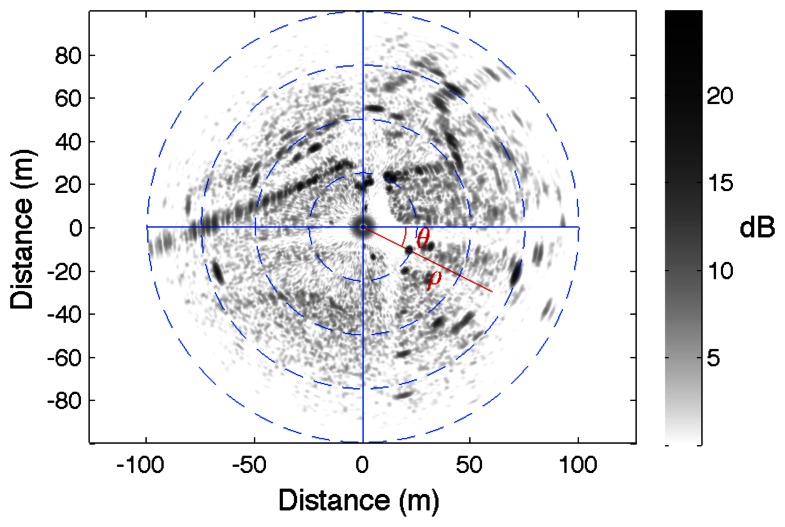
Example of a panoramic radar image. The radar is positioned at the center of the image.

An example of radar image is presented in Figure 2. The radar is positioned at the center of the image. The gray scale level indicates the amplitude of the backscattered signal. Each element of the image is positioned through its polar coordinates (*d*, *θ*).

When the target is moving (see Figure 1(b)), considering the modulation slope, the shift introduced by the Doppler effect is added (negative slope) or subtracted (positive slope). Let us denote respectively *f_bu_* and *f_bd_* the beat frequencies of up and down modulation slopes:

$$\begin{array}{c} f_{bu}=\frac{2R}{c}\alpha -\frac{2\dot{R}}{\lambda }\\ f_{bd}=\frac{2R}{c}\alpha +\frac{2\dot{R}}{\lambda } \end{array}$$

where *Ṙ* is the radial velocity of the detected target, *α* is the frequency step of the modulation. The first part of the equation is linked to the time shift introduced by the distance while the second part is due to Doppler effect.

Based on these equations, the range *R* is calculated by adding *f_bu_* and *f_bd_*:

$$R=\frac{c}{4\alpha }(f_{bu}+f_{bd})$$

The radial *Ṙ* speed is obtained by subtracting *f_bu_* from *f_bd_*:

$$\dot{R}=\frac{\lambda }{4}(f_{d}-f_{bu})$$

So, both the range and the radial velocity are simultaneously estimated with this sensor.

The IMPALA sensor provides two radar images related to the up and down modulation respectively. In the first case, the Doppler effect operates in an additive way and it is the opposite for the second case. It is also important to remind that the Doppler velocity is not only the result of the moving objects in the environment but is linked to the displacement of the vehicle itself. The vehicle's movement during the measurement acquisition from the rotating sensor is a source of disturbance: the panoramic images include a distortion phenomenon. Our goal is to analyze these effects in an odometric purpose.

## 3. Robot's Own Velocity Estimation through the Analysis of Doppler Effect

The Doppler effect is the frequency shift between the emitted and received signals when the distance between emitter and receiver is modified during the acquisition time. It is easy to demonstrate that for an emitter (or receiver) moving at velocity *V* in the direction of the receiver (emitter respectively), emitting at frequency *F*, the frequency modification *F_d_* is given by Equation (1). In case the movement is not in the direction of the receiver, radial velocity has to be considered, so:

(2) $$F_{d}=2\times \frac{V\times \cos(\theta )}{\lambda }\text{with}\;\theta \in [0,2\pi ]$$

By measuring *F_d_* for different directions *θ* (the angle between current measurement and the direction of motion of the radar sensor mounted on the vehicle), radial velocity can be estimated. It is reminded that Doppler effect is produced from the vehicle's own displacement and also from the moving objects in the surroundings. In order to extract the robot's own velocity, global coherence of the surroundings is required, so an assumption is made that more than 50% of the environment is static. On the contrary, if more than 50% of detections are related to mobile objects that have the same displacement, then our estimation is disturbed. For each radar beam, both the up and down modulations are compared in order to extract the Doppler shift using a correlation of each spectrum. From this shift, radial velocity is obtained for each ray of the observation. As radial velocity is a projection of global velocity in each observed direction, velocity profile looks like a cosine function from which parameters have to be estimated:

$$V_{\text{Doppler}}=V(t)\times \cos(\theta )$$

where *V*(*t*) is the velocity of the radar bearing robot during the panoramic acquisition.

Let us denote the robot's velocity profile *V*(*t*) with a polynomial function of the time *t*:

(3) $$\begin{array}{c} V(t)=X(1)\times t^{m}+X(2)\times t^{m-1}+\ldots +X(m+1)\\ \left[\begin{array}{c} V_{\text{Doppler}\;1}\\ \vdots \\ V_{\text{Doppler}\;n} \end{array}\right]=([t^{m}\cdots 1]X)\;\circ \;[\begin{array}{c} \cos(\theta_{1})\\ \vdots \\ \cos(\theta_{n}) \end{array}] \end{array}$$

where ∘ is the Hadamard product function [22].

**Figure 3.**
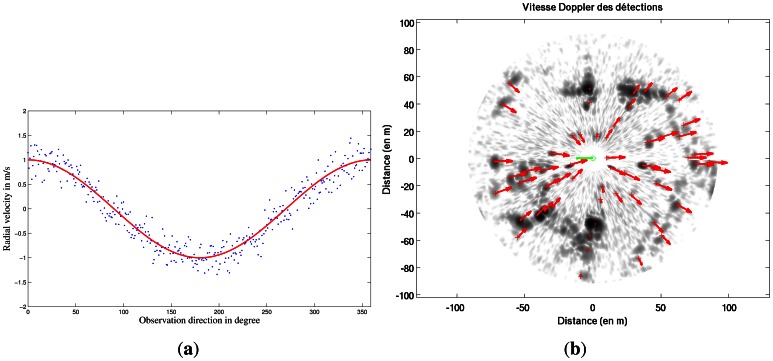
(**a**) Doppler velocity profile estimation during the acquisition; (**b**) Doppler image based on velocity profile. Each red arrow is the Doppler velocity of the detected targets.

Median Least Square algorithm [23] is applied to estimate the parameters of *X* of the function *V*(*t*) based on Doppler estimates for each radar beam. This principle is illustrated in Figure 3(a). Each measurement of Doppler velocity *V_Doppler i_* has an uncertainty *σ_Doppler_*. As a result, parameters of *X* of the function *V*(*t*) are estimated with their own uncertainty. Vehicle's own velocity profile *V*(*t*) and uncertainty *σ_V_*_(_*_t_*_)_ can be known during the radar acquisition.

A Doppler image representing the Doppler effect created by the vehicle is obtained based on the previous estimated robot's velocity profile and Equation (3). This result is presented in Figure 3(b).

## 4. Distorsion Analysis Based Velocimetry

### 4.1. Distortion Formulation

**Figure 4.**
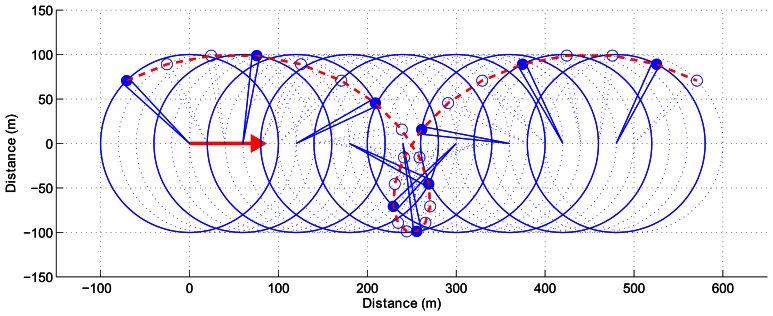
Representation of a simple trochoid described by the sensor beam in the case of a straight line movement.

Data distortion results from combined sensor and vehicle movements. In fact, the displacement of the radar beam during an entire revolution of the sensor can be compared to the movement of a bicycle valve for a simple movement in a straight line (*cf.* Figure 4).

As regards some other displacements of the center of rotation, the distortion equation can be represented by the parametric equation of a trochoid. Indeed, at time *t*, the position of a detection done at range *ρ* is a function of the center pose $\left(x_{t}^{c},y_{t}^{c},\phi_{t}^{c}\right)$ and of sensor bearing *θ_t_*.

(4) $$\left[\begin{array}{c} x(t)\\ y(t) \end{array}\right]=\underset{\text{A})\;\text{Center position at time}\;t}{\underset{︸}{\left[\begin{array}{c} x_{t}^{c}\\ y_{t}^{c} \end{array}\right]}}+\underset{\text{C})\;\text{Detection position time}\;t}{\underset{︸}{\underset{\text{B})\;\text{Center rotation}}{\underset{︸}{\left[\begin{array}{cc} \cos(\phi_{t}^{c}) & -\sin(\phi_{t}^{c})\\ \sin(\phi_{t}^{c}) & \cos(\phi_{t}^{c}) \end{array}\right]}}\left[\begin{array}{c} \rho \cos(\theta_{t})\\ \rho \sin(\theta_{t}) \end{array}\right]}}$$

In order to obtain the center pose at time *t*, an evolution model taking into account the linear (*V*) and angular (*ω*) velocities is formulated. Pose $\left(x_{t}^{c},y_{t}^{c},\phi_{t}^{c}\right)$ is obtained as follows:

(5) $$\{\begin{array}{l} \left[\begin{array}{l} x_{t}^{c}\\ y_{t}^{c} \end{array}\right]=\frac{2V}{\omega }\sin\left(\frac{\omega t}{2}\right)\left[\begin{array}{c} \cos\left(\frac{\omega t}{2}\right)\\ \sin\left(\frac{\omega t}{2}\right) \end{array}\right]\\ \phi_{t}^{c}=\phi_{0}^{c}+\omega t. \end{array}$$

**Figure 5.**
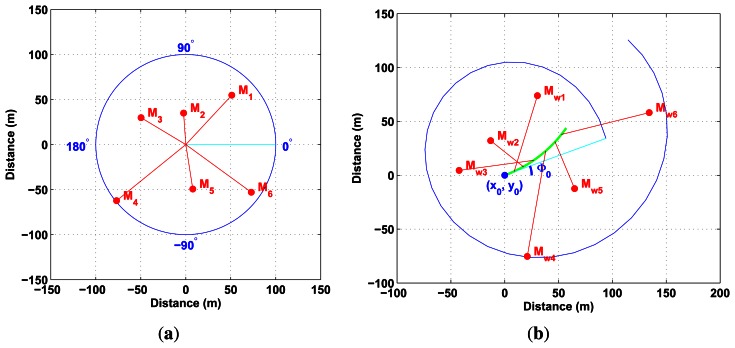
(**a**) Data obtained in sensor frame without considering distortion; (**b**) Undistorted data based on distortion formulation.

Using this distortion equation, which is parametrized with respect to the linear and angular velocities *V* and *ω*, both the distorted or undistorted measurements could be managed. With a prior estimate of these parameters, detection (*θ*, *ρ*), done at time *t* in the sensor frame, can be transformed into the world frame based on Equations (4) and (5) (*cf.* Figure 5).

**Figure 6.**
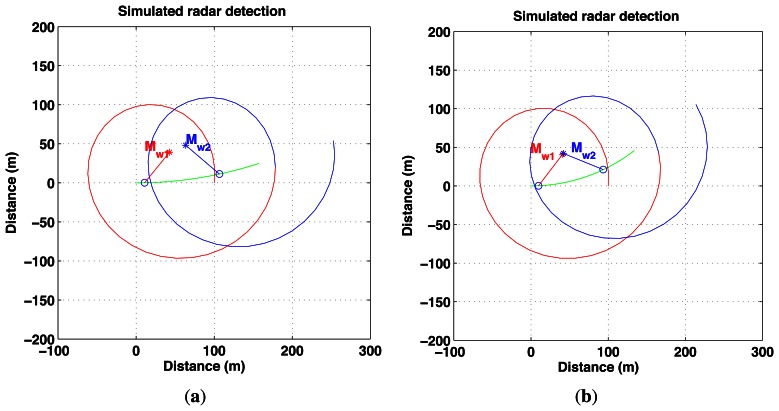
(**a**) Projection of data in world frame with wrong velocity estimates; (**b**) Projection of data in world frame with correct velocity estimates.

**Figure 7.**
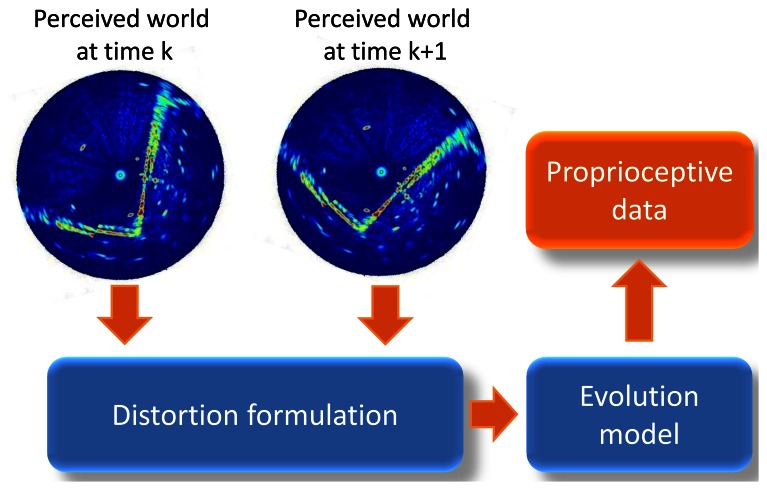
Proposed reverse proprioceptive data estimation based on distortion measurements.

The objective is to estimate proprioceptive information, in fact velocities *V* and *ω*, which best undistort the measurements. Two successive observations are required as shown in Figure 6. This principle is presented in Figure 7.

**Figure 8.**
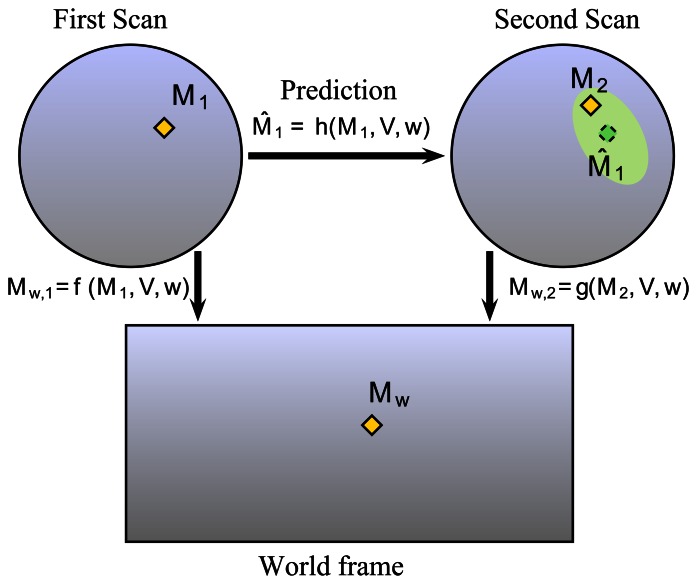
Principle of distortion analysis: the detected landmark in each scan in yellow, and the corresponding landmark in the real world; the predicted detection pose from scan 1 onto scan 2 in green.

In order to extract the information from the distortion phenomenon using a rotating sensor without any knowledge of the environment shape, the required assumption is the local constant velocity of the vehicle during two successive measurements. The pose of each measurement is directly linked to the observation pose and to the angle of observation. This pose can be expressed with the constant velocity model of the vehicle and is only a function of the linear and angular speed of the robot (see Equation (5)). Let M_1_ and M_2_ be the landmarks representing the same point in the world M*_w_* in their respective distorted scans or radar images. It is possible to transform M_1_ and M_2_ into the undistorted world frame by using the parameters (*i.e.*, linear velocity *V* and angular velocity *ω*) and distortion functions *f* and *g* (*cf.*Figure 8). By comparing the different projected poses in each acquisition, velocity parameters can be extracted. In order to achieve this task, data association between images 1 and 2 is required. The prediction function *h* = *g*^−1^ ○ *f* is unknown because *g*^−1^ cannot be obtained; consequently, minimization techniques have to be used in order to estimate Mˆ_1_. Finally, each association can give new values of the velocity parameters.

The sensor on the robot moves from an initial pose x_0_ = [*x*_0_, *y*_0_]*^T^* with an initial orientation *ϕ*_0_ at a constant velocity **V***_υ_* = [*V*, *ω*]*^T^* during two successive sensor scans. Each detection of landmark m*_d_* observed at time *t_d_* is distorted by the robot's displacement. At this step, the detected landmark m*_d_* needs a correction in order to take into account the Doppler effect. If m*_d_* = [*x_d_*, *y_d_*]*^T^* is the perturbed detection of landmark m*_i_* = [*x_i_*, *y_i_*]*^T^* at time *t_d_*, the correction is obtained as follows:

(6) $${\text{m}}_{i}=\left(\sqrt{x_{d}^{2}+y_{d}^{2}}+2\alpha \frac{V\;\text{cos}(\omega_{\text{sensor}}t_{d})}{\lambda }\right)\times \left[\begin{array}{c} \text{cos}(\omega_{\text{sensor}}t_{d})\\ \text{sin}(\omega_{\text{sensor}}t_{d}) \end{array}\right]$$

where *λ* is the wavelength of the radar signal, *α* a coefficient which links frequency and distance, *ω_sensor_* the rotating rate of the sensor. If no Doppler effect has to be considered, as is the case with laser sensors, just note that m*_i_* = m*_d_*. This correction is applied to the whole set of detections M_1_ and M_2_ in the successive scans.

So, M_1_ and M_2_ detected in their respective scans can be propagated in the world frame by their two respective propagation functions:

(7) $$\{\begin{array}{l} {\text{M}}_{w,1}=f({\text{M}}_{1},V,\omega )\\ {\text{M}}_{w,2}=g({\text{M}}_{2},V,\omega ) \end{array}$$

For the first radar image, the function f can be expressed as:

(8) $${\text{M}}_{w,1}=x_{0}+\left[\begin{array}{cc} \cos(\phi_{0}+\omega t_{1}) & -\sin(\phi_{0}+\omega t_{1})\\ \sin(\phi_{0}+\omega t_{1}) & \cos(\phi_{0}+\omega t_{1}) \end{array}\right]\;{\text{M}}_{1}+\frac{2V}{\omega }\sin\left(\frac{\omega t_{1}}{2}\right)\;\left[\begin{array}{c} \cos\left(\phi_{0}+\frac{\omega t_{1}}{2}\right)\\ \sin\left(\phi_{0}+\frac{\omega t_{1}}{2}\right) \end{array}\right]$$

with $t_{1}=\frac{\arctan(y_{1},x_{1})}{\omega_{\text{sensor}}}$.

Similarly, for the second scan M*_w,_*_2_ = *g*(M_2_, *V*,*ω*) can be easily deducted with $t_{2}=\frac{\arctan(y_{2},x_{2})+2\times \pi }{\omega_{\text{sensor}}}$. The function arctan is defined on [−π; +π].

The entire set of detections in the world frame can be easily expressed in a matricial form based on Equation (8). Based on these equations we can conclude that distortion is linked to the velocity parameters (*V*, *ω*), to the landmarks in the two successive scans M_1_ and M_2_, to the initial pose of the robot (x_0_, *ϕ*_0_) and to the sensor scanning rate *ω_sensor_*. But, in fact, the only parameters that need to be estimated are the unknown velocities and consequently the current radar pose.

### 4.2. Extraction of Landmarks

**Figure 9.**
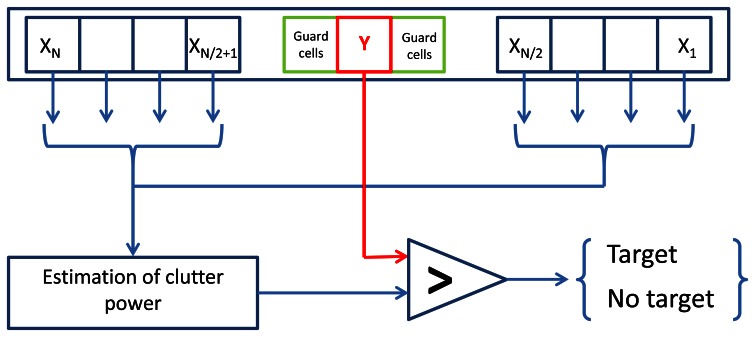
General architecture of CFAR procedures. For a cell *Y*, its direct vicinity is removed (guard cells) and local vicinity *X_1:N_* is processed. If the cell (under test) value is greater than the ponderated local clutter power, the cell is selected as a target.

So far, we defined radar detections *M* as landmarks extracted from the radar data. This extraction is performed using a well-known adaptive threshold technique: the Constant False Alarm Rate (CFAR) detection [24]. CFAR processors are designed to maintain a constant false alarm rate by adjusting the threshold for a cell under test by estimating the interference in the vicinity of the test cell. All the undesired background signals are just denoted as “clutter”. The detection procedure distinguishes between useful target echoes and all possible clutter situations. Clutter is not just a uniformly distributed sequence of random variables, but it can be caused, in practical applications, by a number of different physical sources. A classical Order-Statistic technique (referred to as OS-CFAR) is described in [25]. In OS-CFAR, the threshold is calculated by estimating the level of the noise around the cell under test. As the power from the tested cell can corrupt the average estimate, the direct area around the tested cell is not considered. The size of this area is determined based on the antenna characteristics and the impulse response function of the used radar system. The principle of CFAR processor is illustrated in Figure 9, and in our case we extend this principle in two dimensions by considering not only a single radar power spectrum, but also the entire radar image, across cells of differing bearing angle at constant range.

As ground radar clutter is difficult to model, CFAR processor still provides wrong landmark detections that we need to filter out during estimation of velocities.

### 4.3. Estimation of Velocities

In order to estimate the velocity parameters [*V*, *ω*]*^T^*, the data association between landmarks from the two successive scans has to be done. Mˆ_1_ has to be predicted from M_1_ (in the first scan) onto the second scan. A minimization technique is applied in order to calculate the function Mˆ_1_ = *h*(M_1_, *V*, *ω*) because *h* cannot be calculated directly. The cost function for one landmark is given by *S* = (M*_w_*_,2_ − M*_w,_*_1_)^2^ or:

(9) $$S({\hat{x}}_{1},{\hat{y}}_{1})={\left(g({\hat{\text{M}}}_{1},V,\omega )-f({\text{M}}_{1},V,\omega )\right)}^{2}$$

A gradient method with adaptive step-sizes is used to minimize this cost function. As a result, the prediction of first radar image landmarks can be computed in the second image as well as its uncertainty ellipsis. Data association between prediction Mˆ_1_ and landmark M_2_ is then calculated based on Mahalanobis distance criteria by taking into account uncertainties of measurements and predictions.

As radar data are very noisy and ground clutter is difficult to estimate, both landmark extraction and data association can be false. For example, the speckle effect can lead to ghost detections or false disappearances due to the different possible combinations of radar signals. Moreover, due to multiple reflections, radar data are not as accurate as laser data. Thus, firstly, all possible data associations have to be considered. Then a filtering method has to be applied to filter out wrong associations.

Two assumptions are made at this point. First, the percentage of detections due to static objects has to be sufficient. Indeed detections coming from moving objects can lead to false velocity estimates. Moreover, if all the detections coming from moving objects are coherent and give the same velocity estimate, the most pessimistic requirement is that 50% of the detections in the environment have to come from static objects. Second, the vehicle equipped with the radar sensor is supposed to be moving during two consecutive acquisitions at a constant velocity (*V* and *ω*). Actually, when the vehicle accelerates or decelerates, the estimated velocity obtained will be the mean speed of the vehicle.

For each possible data association allowed by the Mahalanobis distance, a new estimate of the robot's velocity is computed and sent over to an Extended Kalman Filter process. Then, for this association assumption, updated speeds are projected into the velocity space with their respective uncertainties. As a result, in this space, each association assumption votes for the robot's velocity. Then, the global coherence of the scene is researched by a RANSAC process [26]. This operation permits to discard wrong detections and associations due to non-coherent velocity estimates.

At this step, we suppose that most of the remaining detections are static and well associated. The final fusion of estimated velocities uses the Covariance Intersection (CI) method [27] in order to remain pessimistic in the case of any residual wrong vote during fusion.

To ensure a robust process, both CFAR processor and RANSAC technique are used to minimize the number of wrong associations. The entire process is briefly summarized in Algorithm 1.


**Algorithm 1** Odometry algorithm based on rotating range sensor.
 **INPUT:**
-2 successive radar scans-the last estimated robot velocities: (*V*, *ω*) M_1_ ← CFAR detections from scan 1 M_2_ ← CFAR detections from scan 2 Prediction of detections from image 1 onto image 2:
-Mˆ_1_ ← arg min{(*g*(Mˆ_1_, *V*, *ω*) − *f*(M_1_, *V*, *ω*))^2^} AssumptionAsso ← Data association phase between M_2_ and Mˆ_1_ **for**
*k*: AssumptionAsso **do**   **Vˆ**_v_(*k*) ← Extended Kalman Filter update *k* (*V*, *ω*) **end for** **Ṽ**_inlier_ ← RANSAC filtering of velocities assumptions **Vˆ**_v_ **Ṽ**_v_ ← CI Fusion (**Vˆ**_inlier_) **OUTPUT:** new estimated robot velocities *Vˆ_υ_* = [*V*, *ω*]*^T^*


## 5. Results and Discussion

**Figure 10.**
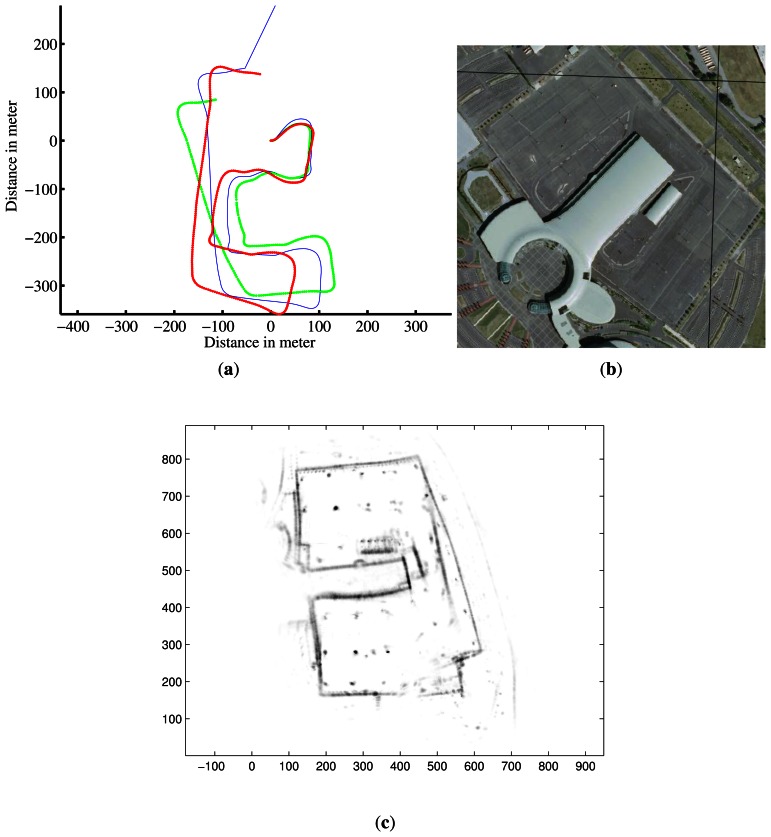
Trajectory and map reconstruction based on IMPALA radar odometry. (**a**) Estimated trajectory with IMPALA odometry: in blue the ground truth, in red the reconstructed trajectory with the measurements of the odometer and gyrometer, and in green radar-odometry solution; (**b**) Aerial view of the experimental area; (**c**) Map obtained based on radar odometry with the estimated velocities.

**Figure 11.**
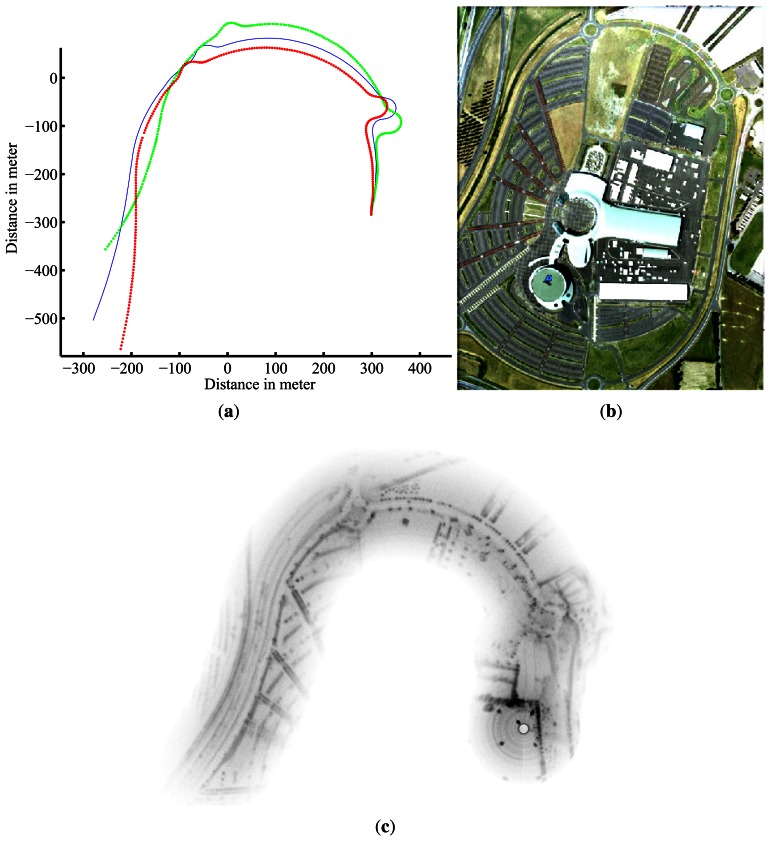
Trajectory and map reconstruction based on IMPALA radar odometry. (**a**) Estimated trajectory with IMPALA odometry: in blue the ground truth, in red the reconstructed trajectory with the measurements of the odometer and gyrometer, and in green radar-odometry solution; (**b**) Aerial view of the experimental area; (**c**) Map obtained based on radar odometry with the estimated velocities.

**Figure 12.**
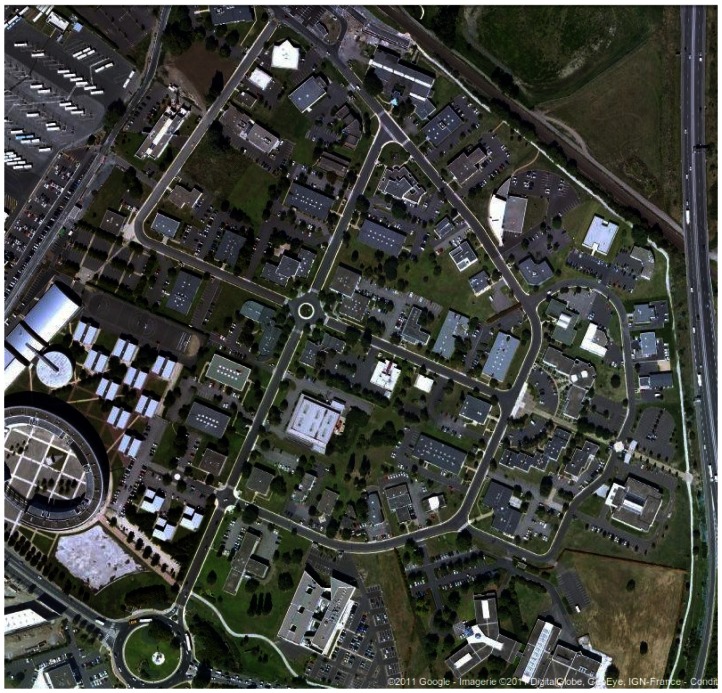
Aerial view of the experimental area: *La Pardieu*, Clermont-Ferrand, FRANCE.

This section provides experimental results of the presented radar-based approach. The IMPALA radar sensor was mounted on a utility car, on top of the vehicle, 2 meters above the ground. The experimental runs that are presented were conducted in an outdoor field, near Clermont-Ferrand in FRANCE, on Blaise Pascal University campus and around the Auvergne Zenith car-park (*cf.* aerial view in Figures 10(b), 11(b) and 12), with a semistructured environment (buildings, trees, roads, road signs, *etc.*). Speed estimation has been done on different kinds of displacements, *i.e.*, rectilinear displacement and also classical road traffic displacement with different curves.

In order to evaluate the performance assessment of the approach, a DGPS system and an odometer sensor were used to provide a reference trajectory and to fuse their measurements in order to estimate the vehicle's velocity. Radar images were recorded and post-processed as explained previously.

### 5.1. Doppler Velocimetry

**Figure 13.**
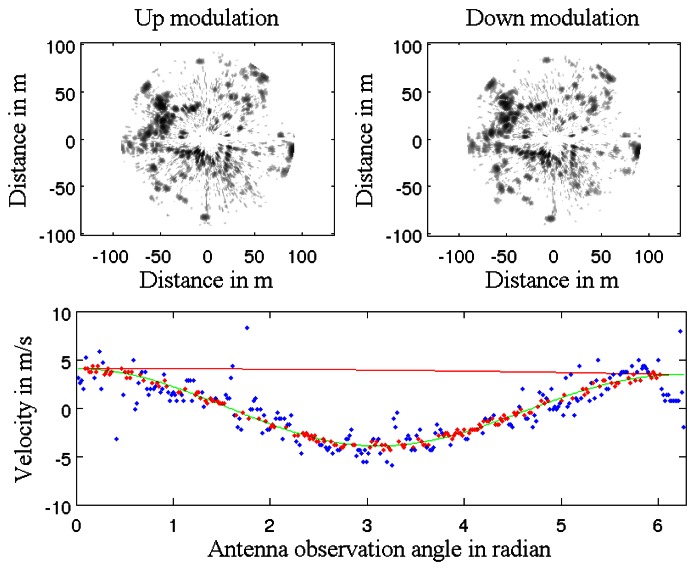
Robot's velocity profile estimation step: top left, up radar image, top right, down radar image. Extracted Doppler velocity and robot's velocity profiles in green and red lines respectively.

As a first step, the robot's own velocity has been estimated with different data sets acquired from the IMPALA radar sensor. The results of velocity profile extraction based on the method described in Section 3 are presented in Figure 13: on top, the two radar images obtained with the up and down modulation during a single antenna rotation. For each acquired radar beam, velocity is estimated (in blue dots) based on correlation techniques. The median least square method using covariance of the extracted Doppler is used to select inliers Doppler detection (in red dots) and to process the robot's velocity profile during the acquisition (in red line). The Doppler velocity profile is estimated in green line.

**Figure 14.**
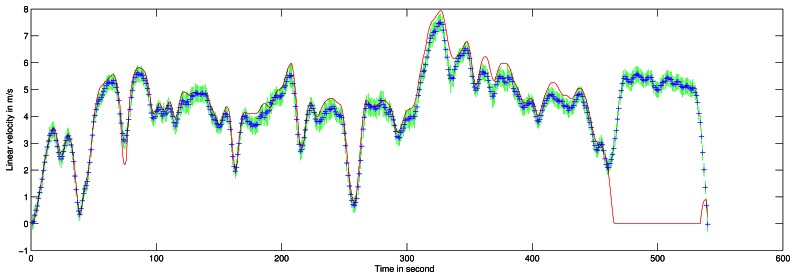
Robot's velocity profile estimation during the entire acquisition based on Doppler effect analysis. In red, ground truth velocity is obtained with filtered odometer data. In blue are the estimates given by the method with the associated 1 *σ* uncertainty in green.

**Figure 15.**
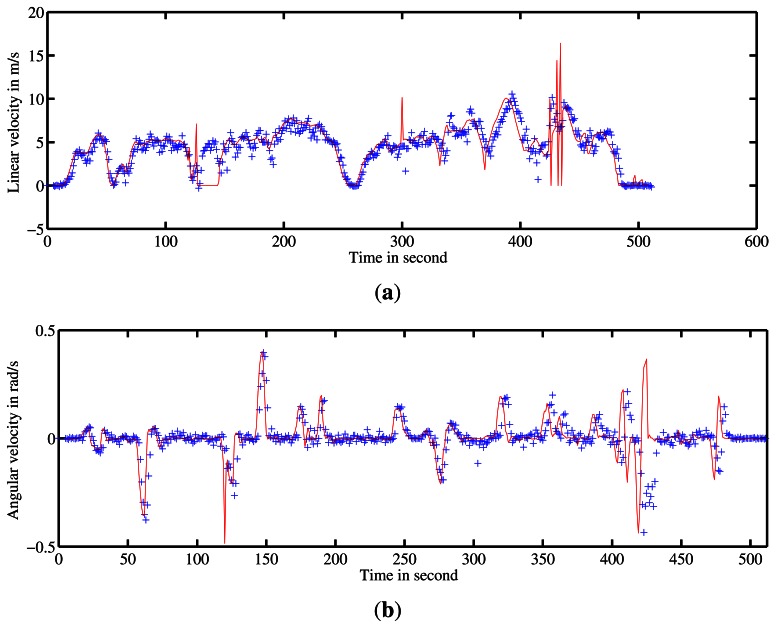
Estimation of velocities by the IMPALA radar-based odometry approach. (**a**) and (**b**) represent the linear and angular velocity estimates respectively. Ground truth velocity obtained by D-GPS and odometer is in red. In blue are the estimates given by the method.

The robot's velocity obtained during a 10-minute 2-kilometer travel is presented in Figure 14. Maximum speed during this travel was approximately 30 *km*/*h*. Trajectory is presented on aerial image in Figure 15. Ground truth for velocity is taken from filtered odometer data. The acquisition system encountered a problem at the end of the experiment, so no reference is available for the last few meters.

Doppler velocity estimation with correlation presents a standard deviation of 0.3 *m*/*s* which corresponds to the correlation resolution. The estimated speed with its respective uncertainty is presented in Figure 13. A statistical evaluation of our Doppler odometry has been performed. The linear velocity estimate error ∊*_V_* has a standard deviation *σ_∊V_* = 0.76 *m*/*s* and a mean ∊̄*_V_* = 0.27 *m*/*s*. An error during the classical odometer recording occurred at the end of the trajectory, which explains the 0 values on the red data while Doppler is still estimating the velocity.

### 5.2. IMPALA Radar-Based Odometry and Map Reconstruction

It has been shown that the linear velocity of the vehicle can be estimated from a complete revolution of the panoramic IMPALA radar sensor. But in order to localize the robot, both the angular and linear velocity needs to be estimated. Then an evolution model can be used to infer the current pose of the vehicle.

The angular and linear velocities are estimated by the proposed distortion-based approach (*cf.* Section 4). Coupled with the robot's own velocity estimation through the analysis of Doppler effect, these observations can be used in order to recover the displacement parameters.

In order to demonstrate the efficiency of our approach, various runs were conducted, first in traffic-free environments over a 2.5-kilometer travel at a mean speed of 6 *m*/*s*, then a 1.4-kilometer travel at 4 *m*/*s* and finally over a 1.5-km travel at 5 *m*/*s*. Then, in order to evaluate the robustness of the approach to dynamic environment, different experiments (for a total of 3 kilometers) are proposed in dense traffic conditions on *La Pardieu*, Technological Park in Clermont-Ferrand, France.

#### 5.2.1. Experiments on Blaise Pascal University Campus

A first run was conducted on Blaise Pascal University campus. The utility car was driven over 2.5-km at a mean speed of 6 *m*/*s*. Angular and linear velocities were estimated during the entire trajectory. These estimates were compared with ground truth. The results of velocity estimation are presented in Figure 15.

**Figure 16.**
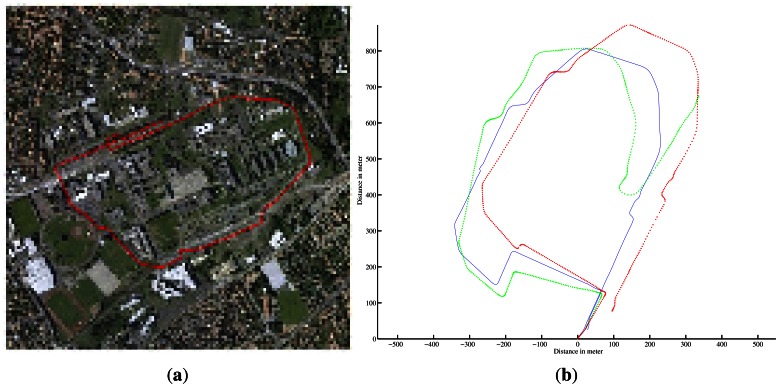
Localization results with radar-based odometry. (**a**) The D-GPS ground truth. (**b**) In blue, the ground truth; in red, the vehicle localization based on dead reckoning with the proprioceptive sensors; in green, the result with radar-based odometry.

The trajectory inferred by the velocities and the ground truth trajectory are both presented in Figure 16. One can notice errors when the vehicle is turning at 90° and a divergence of the algorithm at the end of the trajectory (in green) can be seen. The errors in the velocity estimates can be explained by the fast variation of the vehicle's orientation. In such conditions, the assumption of constant velocity required by the algorithm is not respected. Similarly, by default, the radar-odometry algorithm produces sharp curves where the angular velocity changes from −0.2 *rad*/*s* to 0.2 *rad*/*s*. As a result the algorithm cannot converge.

#### 5.2.2. Mapping Experiments on Auvergne Zenith Car-Park

**Figure 17.**
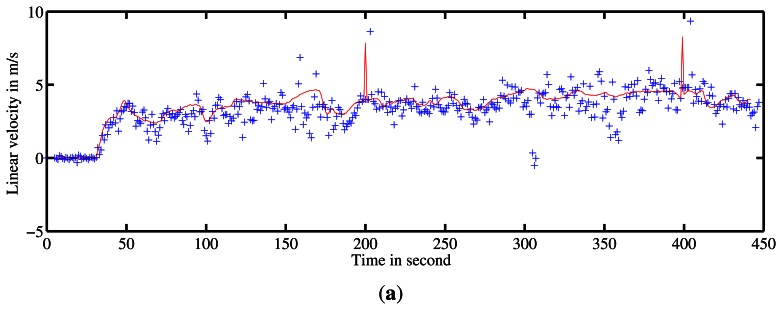

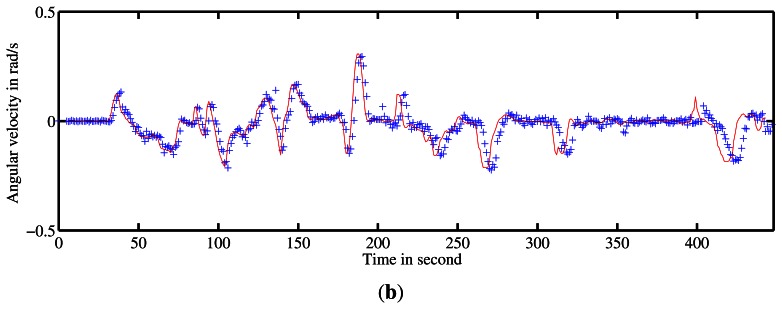
Estimation of velocities. (**a**) and (**b**) represent the linear and angular velocity estimates respectively. Ground truth velocity is in red. In blue the estimates given by the method are shown.

**Figure 18.**
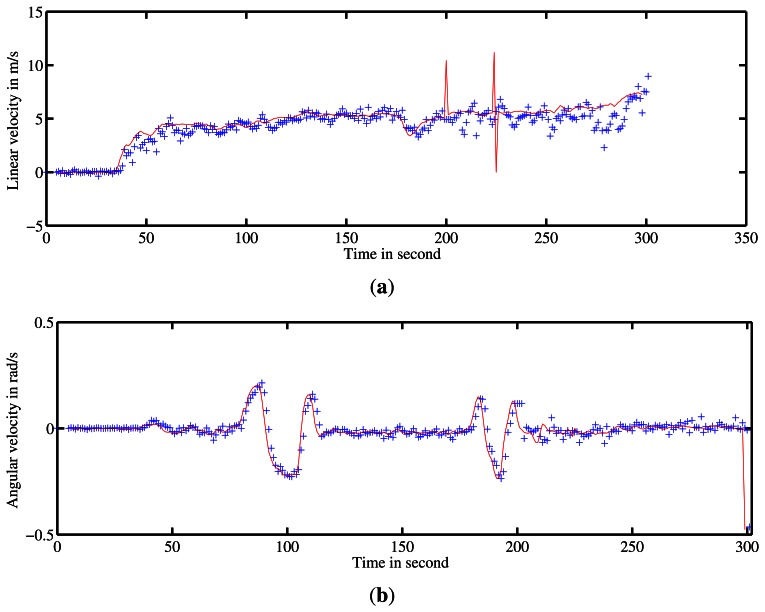
Estimation of velocities. (**a**) and (**b**) represent the linear and angular velocity estimates respectively. Ground truth velocity is in red. The estimates given by the method are in blue.

Two other experiments were conducted on Auvergne Zenith car-park. The vehicle was driven over trajectories of 1.4-km and 1.5-km at a mean speed of 5 *m*/*s* in a static environment. The linear and angular velocity estimates are presented in Figures 17 and 18 respectively.

For the first run, the trajectory and the map obtained based on an evolution model and velocity estimates are presented in Figure 10. Once again, divergence can be observed at 90° curve due to the violation of the assumption of constant velocity.

Similarly, trajectory and mapping results for the second test are presented in Figure 11.

#### 5.2.3. Experiments in Dynamic Environment

In order to evaluate our algorithm in dynamic environment, experiments were conducted in dense traffic conditions in *La Pardieu*, Technological Park in Clermont-Ferrand, France. An aerial view of the experimental area is presented in Figure 12.

**Figure 19.**
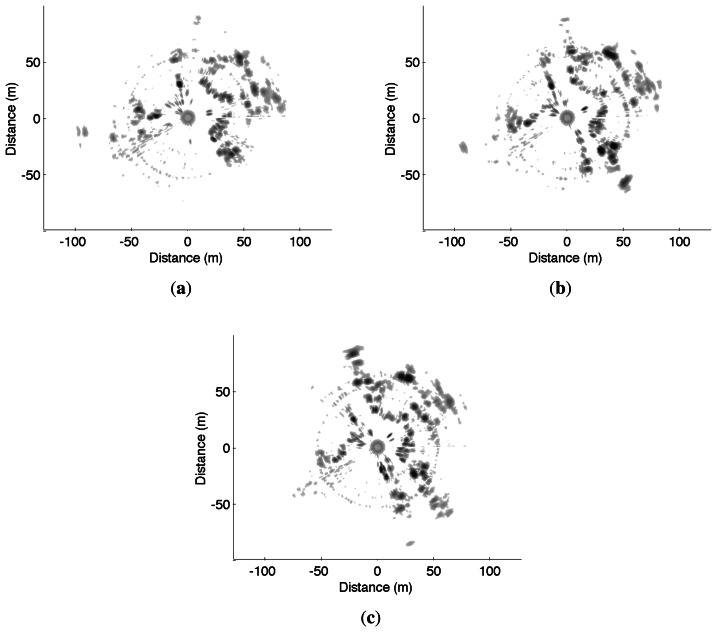
Three consecutive IMPALA radar images used for odometric and mapping purpose: (**a**) *t* = 100 *s*; (**b**) *t* = 101 *s*; (**c**) *t* = 102 *s*.

An example of three consecutive radar images acquired during these experiments is presented in Figure 19. Visual interpretation of the displacement between these radar scans is difficult, even for a human operator, whereas the proposed approach, coupling both Doppler analysis and distortion evaluation, gives promising results.

Different trajectories were executed at a mean speed of 4 *m*/*s* in this dynamic environment. For each experiment, velocity estimates, localization result with ground truth and mapping are presented.

During these experiments, very few landmarks are detected in successive radar images. The number of used landmarks is around 0 to 15 with a mean of 10 reliable detections. If no landmark is detected, the pose prediction is only based on the evolution model with constant velocity.

The quantitative evaluations related to the trajectories and the velocity estimates have been conducted in the same way as the previous experiments and are presented in Table 2.

## 6. Conclusions

An original method for the computing pose and instantaneous velocity of a mobile robot in natural or semi-natural environments was presented using a slow rotating range sensor and considering both the data distortion involved and the Doppler effect. The distortion formulation due to the displacement of the sensor was established. Comparison techniques between successive scans were applied to obtain the robot's angular and linear velocity parameters. Even under the assumption of constant velocity, the algorithm is robust at moderate velocity variations. The sensor used for this study was a panoramic radar sensor, with Doppler effect consideration, but the general formulation can easily be adapted to other rotating range sensors. With such a kind of ground-based radar sensor, the extraction and processing of landmarks remain a challenge because of detection ambiguity, false detection, Doppler and speckle effects and the lack of detection descriptors. In order to deal with these problems, radar signal processing and a voting method were implemented. The approach was evaluated on real radar data showing its feasibility and reliability at high speed (≈30 km/h). The main novelties of the proposed approach include considering distortion and Doppler effect as sources of information rather than as disturbances, using no other sensor than the radar sensor, and working without any knowledge of the environment.

## Figures and Tables

**Figure 20.**
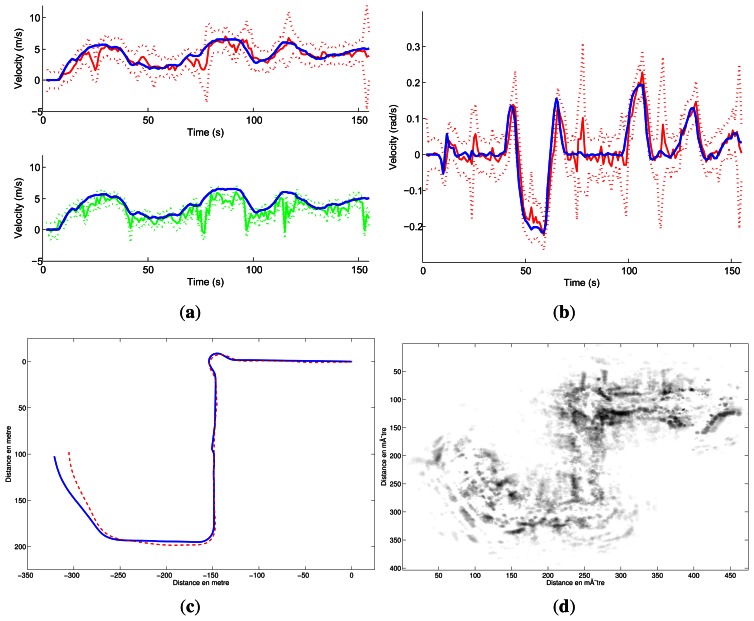
IMPALA odometry results in dynamic environment. (**a**) Linear velocity estimation: in blue the ground truth plotted on the two graphs, in green the Doppler estimation and in red the odometry estimation, both plotted with their respective uncertainty; (**b**) Angular velocity estimation (in red) along with ground truth; (**c**) Estimated trajectories: in red the IMPALA odometry, in blue the ground truth; (**d**) Map obtained based on the trajectory solution.

**Figure 21.**
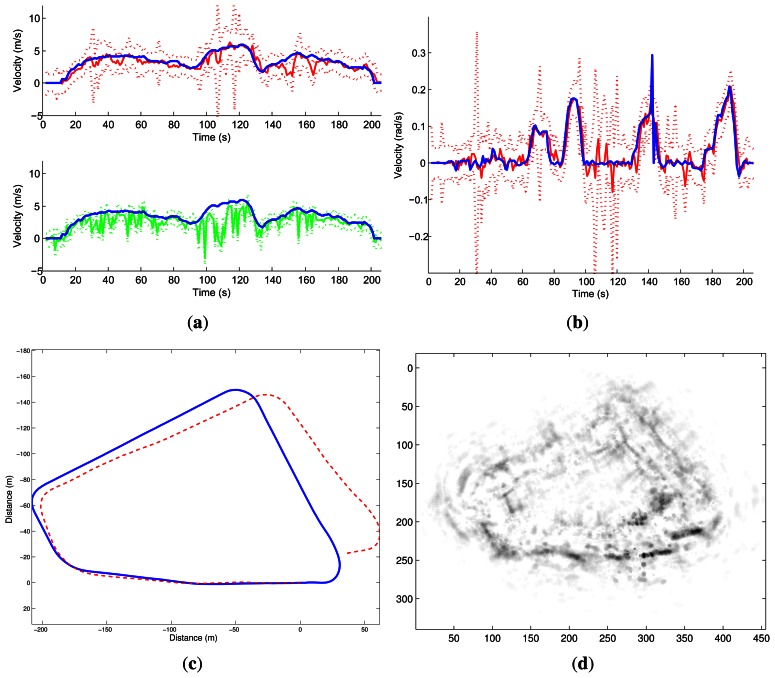
IMPALA odometry results in dynamic environment. (**a**) Linear velocity estimation: in blue the ground truth plotted on the two graphs, in green the Doppler estimation and in red the odometry estimation, both plotted with their respective uncertainty; (**b**) Angular velocity estimation (in red) along with ground truth (**c**) Estimated trajectories: in red the IMPALA odometry and in blue the ground truth; (**d**) Map obtained based on the trajectory solution.

**Table 1.** Characteristics of the IMPALA radar.

Transmitter power *Pt*	20 dBm
Antenna gain G	20 dB
Range	3 m/100 m
Carrier frequency *F*0	24.125 GHz (K band)
Angular resolution (horizontal)	4°
Distance resolution *δR*	1m
Velocity resolution *δV*	0.6 m/s
Size (length-width-height)	29-24-33 cm
Weight	10 kg

**Table 2.** Ego-motion results with the IMPALA radar.

**Experiments**	Figure 20	Figure 21
∊*_x_* (in m)	0.1191	−0.1781
*σ_x_* (in m)	1.8560	0.8714

∊*_y_* (in m)	−0.3605	0.2138
*σ_y_* (in m)	1.1951	0.7726

∊*_θ_* (in rad)	−0.0771	0.1118
*σ_θ_* (in rad)	0.1902	0.3839

∊*_V_* (in m/s)	0.1394	0.3702
*σ_V_* (in m/s)	0.9855	0.8282

∊*_w_* (in rad/s)	−0.00016	0.00014
*σ_w_* (in rad/s)	0.0506	0.0435
